# The effects of individual and community-level factors on maternal health outcomes in Ghana

**DOI:** 10.1371/journal.pone.0207942

**Published:** 2018-11-29

**Authors:** Joseph Adu, Eric Tenkorang, Emmanuel Banchani, Jill Allison, Shree Mulay

**Affiliations:** 1 Division of Community Health and Humanities, Faculty of Medicine, Memorial University of Newfoundland, St. John's, NL, Canada; 2 Department of Sociology, Faculty of Humanities and Social Sciences, Memorial University of Newfoundland, St. John’s, NL, Canada; Paediatric Centre of Excellence, ZAMBIA

## Abstract

**Background:**

Utilization of maternal health care services is key to reducing the number of perinatal deaths and post-natal complications in sub-Saharan Africa. With a few exceptions, many studies that examine the use of maternal health services in sub-Saharan Africa have focused largely on individual-level explanations and have ignored the importance of contextual and community-level explanations. In Ghana, progress has been made in reducing maternal mortality ratio from 740/100,000 in the late 1990s to 319/100,000 in 2015 but these rates are still high. Our study focuses on impact of individual and community level-factors on maternal outcomes with the hope that it will inform public policy in Ghana. This approach highlights latent or unacknowledged aspects of fragility within health systems designed to improve maternal health and opportunities for improving uptake of services.

**Methods and findings:**

Using the 2014 Ghana Demographic and Health Survey, we examined the effects of individual and community-level factors on antenatal care, facility-based delivery, and post-natal care. Multilevel logistic regression models were used to examine the effects of individual and community-level factors on the outcome variables. Our analysis revealed that overall utilization of antenatal, facility-based delivery and post-natal care was substantial across the board; however, both individual and community-level factors were significant predictors of these maternal health outcomes. Wealthier and better educated women were more likely to use antenatal services and facility-based delivery; in contrast poor and uneducated women were more likely to use antenatal and postnatal care but not facility-based delivery. Additionally, use of National Health Insurance Scheme was statistically associated with the utilization of maternal health services.

**Conclusions:**

The findings point to areas where services can be better tailored to meet community-specific needs. Policy makers must consider factors such as educational levels and economic security at both individual and community-levels that shape women’s preferences and uptake of maternal health care in Ghana.

## Introduction

Data on maternal mortality in developing countries, especially sub-Sahara Africa (SSA) are disturbing. The World Health Organization (WHO) estimates the maternal mortality ratio (MMR) in SSA is 545/100,000 live births [1], this alarming statistic is most likely a consequence of lack of obstetric services in poorly-resourced countries. Ghana, one of the countries in SSA has made significant strides by reducing MMR from 740/100 000 live births in the 1990s to 319/100,000 live births in 2015 [2, 3]. Despite Ghana’s progress over the past two decades, some studies suggest that low rate of skilled birth attendants during labor may account for continued relatively high rates of MMR [4, 5]. Skilled birth attendants in this context means either a trained midwife, a nurse, community health nurse, physician assistant or a medical doctor assisting a woman during labor in a health facility. This is coupled with facility-based delivery in a hospital, a health center, a community clinic or a maternity home, but not in the home of the pregnant woman. Inadequate maternal care services, low uptake of these services and inadequate access to antenatal visits, facility-based delivery, and postnatal care have contributed to the present rate of MMR in Ghana [6]. It is well known that improved obstetric care reduces maternal and neonatal mortality rates [7,8].

Many factors influence utilization of maternal care services be it socio-cultural factors [9, 10] or lack of skilled birth attendants; often trained midwives, refuse postings to rural areas [11]. The inequitable distribution of such resources in rural Ghana put women continually at risk during child birth [12]. There have been reports that even tertiary-care hospitals, like the Okomfo Anokye teaching hospital (the second largest hospital in Ghana), lacked operating rooms for emergency Caesarean section (C-section) leaving women in hospital corridors to wait for their turn in the only functional operating room, exposing them to greater risk of poor maternal and neonatal outcomes [13]. Poverty and lack of female education are also strong predictors of maternal health care utilization. Women with less education from poor households are less likely to have access to quality care due to financial and socio-cultural barriers [14, 15]. Moreover, the Community-Based Health Planning and Services (CHPS) which was intended to deliver care at the door steps of the expectant mother and which contributed greatly to the gains made in Millennium Development Goals (MDG) 4 and 5 [16, 17], have gradually been eroded over the years. According to Atuoye et al. [18] and Gething et al. [19], the absence of good transport system, coupled with poor road networks make it difficult for rural women to access facility-based births.

In summary, community interventions targeting antenatal care, identify high-risk pregnancies early so that appropriate measures can be taken to avoid complications during labor [20, 3]. The existing literature shows clear connections between household and community-level socio-economic factors and maternal healthcare utilization in general [21, 22]. Yet, with some exceptions [23, 24, 20], there has been limited assessment of this important relationship. Previous studies that examined utilization of maternal healthcare services in SSA, including Ghana, focused primarily on individual-level factors with little attention to contextual and community-level factors.

This paper fills an important knowledge gap by examining the impact of both individual and community-level factors on the utilization of three maternal health services, namely antenatal care (ANC), facility-based delivery (FBD) and post-natal care (PNC) in Ghana. The findings from this study will be useful to inform policy makers in designing effective intervention and strategies to improve maternal health care. The findings will also help promote evidence-based policy making to address the challenges Ghanaian women face during pregnancy.

## Methods

The 2014 Ghana Demographic and Health Survey (GDHS) was conducted by the Ghana Statistical Service (GSS), Ghana Health Services (GHS); ICF Macro International provided technical assistance through the Demographic Health Survey Program (DHS) [3]. The DHS is a nationally representative cross-sectional survey conducted in developing countries including Ghana. These surveys constitute one of the richest sources of information presently available to examine the determinants of maternal and child health in developing countries.

Data from the 2014 GDHS were used to examine the effects of individual and community-level factors on maternal health services utilization (ANC, FBD and PNC) by women in Ghana. Information from the women’s questionnaire was used for the study. This questionnaire covers detailed information on women in their reproductive years (aged 15–49 years) with live births in the 5 years preceding the survey. The survey uses a two-stage stratified sample frame that allows estimates of key indicators at the national level: urban and rural areas for each of the ten administrative regions in Ghana. A total of 427 clusters, consisting of 216 from urban and 211 from rural areas of Ghana, identified 9,656 women for individual interviews; but only 9,396 women were interviewed, yielding a response rate of 97%. However, the present study is limited to only 4141 women who had their last delivery in the five years preceding the survey and answered questions on utilization of ANC, FBD and PNC services. This means that women who gave birth prior to the survey period and those who did not answer all questions related to antenatal, facility-based delivery and postnatal care were excluded from the analysis. This was to avoid recall bias in the responses of women that could affect the outcome of the study.

Demographic Health Surveys use clustered sampling design to avoid over-sampling or under-sampling of respondents. The 2014 GDHS also used clustered sampling design and applied standardized weights to ensure representative data across all regions including rural areas of Ghana to avoid over-sampling or under-sampling of participants.

### Measures

The three independent variables used in this study were utilization of ANC, FBD, and PNC. The questionnaire specifically asked information on the number of ANC visits, PNC visits and the location of the delivery. Frequency of ANC visits was coded as *‘0’ for less than four (4) visits*, *and ‘1’ for at least four (4) visits*. Although the number of ANC visits depends on the risk status of the expectant mother, WHO recommends at least four visits for the entire period of pregnancy. A visit during the first trimester is recommended for the purposes of screening and identifying infections [3]. The second dependent variable, FBD asked respondents whether the delivery was at home, government hospitals and health facilities, including different types of private health facilities, maternity homes or with traditional birth attendants. Home delivery in this context meant delivery without the assistance of any trained health professional such as a nurse or a midwife. The places of delivery were grouped into two categories; home and FBD and *coded as ‘0’ for home delivery and ‘1’ for FBD*. Skilled delivery during the birthing process is generally considered to be the gold standard since labor and delivery is considered the most critical period of the pregnancy-childbirth continuum. For the third dependent variable, PNC, respondents were asked whether a health care provider had checked them after giving birth or within two months after delivery? The response *was coded as ‘0’ for no and ‘1’ for yes*. Postnatal care for mothers and their newborns is vital as it ensures essential follow-up care for any complications that may arise during or after delivery. It also provides information on how the mother takes care of herself and her newborn [2, 3].

### Independent variables

Independent variables selected for analysis were both at the individual and community levels; namely independent variables at individual and community level, respectively. Individual-level variables tap the socio-economic and demographic characteristics of respondents. *Socio-economic* variables included: educational background of women (coded, no education = 0, primary education = 1, secondary education = 2 and higher education = 3). Wealth status of the household was derived from a combination of assets within the household which included television, bicycle, or car, as well as dwelling characteristics, such as a source of drinking water, sanitation facilities, and type of flooring material and fuel used for cooking. It was computed using principal component analysis (PCA), a multivariate statistical method used to reduce the number of variables in a data set and has been validated as a technique to describe socio-economic status differentiation within a population [25]; the wealth status was coded as low income = 1; middle income = 2 and high income = 3. Employment status was coded as not working = 0 and working = 1). *Demographic and socio-cultural* variables included in the analysis were: *marital status* (never married = 0, married/living together = 1 and divorce/separated/widowed = 2); age of respondent measured in complete years; ethnicity (Akans = 0; Ga/Dangme = 1; Ewes = 2; Northern tribes = 3 and others = 4); religion (Christian = 0; Muslim = 1; Traditionalist = 2 and No religion = 3); residence (urban = 0 and rural = 1) and parity at birth (‘less than 3 births’ = 0; ‘3 to 5 births’ = 1; and ‘6 or more births’ = 2. *Access to health care* was captured with variables that asked respondents if they were registered with the National Health Insurance Scheme (NHIS) (no = 0 and yes = 1) and if distance to the health facility was a problem (not a problem = 0 and a problem (difficulty) = 1).

### Community-level factors

This study used Enumeration Areas (EAs) to represent communities/neighborhoods mainly because the DHS did not collect aggregate-level data at the community/neighborhood level. A total of 427 EAs (216 in urban areas and 211 in rural areas) were selected from the GDHS 2014 survey. Hence, the aggregation of women’s responses to questions at the individual-level was used to create the community-level factors. These responses reflected questions on women’s years of education and wealth status. Community-level variables included in the analysis captured the socio-economic characteristics of the neighborhoods, especially as these have implications for accessing ANC, FBD, and PNC. In brief, the community-level variables were generated by computing the average years of education attained by women in the 427 clusters or communities, and the mean income score was created from the mean values of the wealth index scores of individual women in the various clusters used for survey.

### Analytical technique

Descriptive statistics (means and percentages) were used to examine sample characteristics for women attending ANC and PNC, as well as those who delivered their babies in health care facilities (FBD). Binary logit models were used mainly because of the dichotomous nature of the outcome variables. The values, ‘0’ and ‘1’were used to represent outcomes such as yes/no or < 4 and > 4 ANC visits. For instance, the dependent variables ANC, FBD, and PNC each have two opposing groups (< 4 and > 4 visits; facility and home delivery; PNC and no PNC) respectively. However, these models were computed using a multi-level framework at the individual and community levels. There were two main reasons why multi-level analysis was used in this study. First, it allows the estimation of the significance and magnitude of clustering within the data without which parameter estimates may be biased due to the possible violation of the independence assumption underlying standard logit models [26]. Second, it allows the estimation and explanation of heterogeneity at both the individual and community-levels which is central to this work. Overall, three separate multilevel logit models were estimated for the three outcome variables. The first model estimates the effects of socio-economic predictors on the outcome variables. The second model adds demographic and socio-cultural predictors and the third model, variables that capture access to health care. The statistical package Hierarchical Linear & Non-linear Modeling (HLM) version 7.0 was used to estimate all models [27].

### Ethical consideration

Ethical consent to undertake DHS in Ghana was approved by the Ethics Committee of ICF Macro in Calverton, USA, and the Ethics Committee of Ghana Health Service, Accra, Ghana. Ethics approval for use of this data for the present analysis was obtained from the Ethics Committee of ICF Macro in Calverton, USA through an online application.

## Results

### Descriptive results

Tables 1 and 2 summarize the distribution of independent, individual, and community-level variables. Results show that irrespective of their marital status, majority of women had four or more antenatal visits (89.2%), while very few had less than four visits (10.8%). A large proportion of women had FBD (74.2%) compared to home delivery (25.8%). Moreover, a greater number of the women attended PNC (73.3%) within two months of delivery compared to women who did not receive any care after delivery (26.7%) (See Tables 1 and 2 below for details). In short, irrespective of their educational level, employment status, wealth status, ethnicity, religious affiliation or residential status, large number women used ANC, FBD, and PNC.

**Table 1:** A univariate distribution of selected dependent and independent variables, GDHS 2014 continued.

	N = 4141		
Parameters	Percent	Mean	Standard Deviation
**Dependent variables**			
*Number of Antenatal visits*			
Less than 4 times	10.8		
Four times or more	89.2		
*Place of Delivery*			
Home delivery	25.8		
Facility delivery	74.2		
*Postnatal care up to 2 months after delivery*			
No	26.7		
Yes	73.3		
**Individual level variables**			
**Socio-economic variables**			
*Education*			
No education	31.9		
Primary education	20.1		
Secondary/higher education	47.9		
*Wealth Status*			
Low income	51.1		
Middle income	19.1		
High income	29.7		
*Employment status*			
Not working	20.6		
Working	79.4		

**Table 2:** A univariate distribution of selected dependent and independent variables, GDHS 2014.

	N = 4141		
Parameters	Percent	Mean	Standard Deviation
**Individual level variables**			
**Demographic and socio-cultural variables**			
*Mean age of respondents*		30	7.1
*Ethnicity*			
Akans	38.8		
Ga/Dangme	4.6		
Ewes	11.1		
Northern tribes	43.7		
Others	1.8		
*Religious denomination*			
Christian	72.2		
Muslim	21		
Traditionalists	3.1		
No religion	3.8		
*Marital Status*			
Never married	8.5		
Married/Living together	84.7		
Divorced/Separated/Widowed	6.8		
*Residence*			
Urban	42.1		
Rural	57.9		
*Parity*			
Less than 3	45.3		
3 to 5 births	42.8		
6 or more births	11.9		
**Access to healthcare**			
*Respondent has NHIS *			
No	29.9		
Yes	70.1		
*Distance to health facility (km)*			
Not a problem	69.8		
A problem	30.2		
**Community level variables (N = 427)**			
*Residence*			
Urban	49.4		
Rural	50.6		
Mean years of education (years)		6.9	2.96
Mean income (Ghana cedi)		316.2	829.5

Tables 3 and 4 show bivariate relationships between outcome and predictor variables. It is clear that the educational level of women was significantly associated with ANC and place of delivery. Compared with those with no education, women with secondary/higher education were 76% more likely to make the recommended number of ANC visits (at least four visits), and 29% more likely to opt for health facilities for delivery services. In general, respondent’s wealth index was statistically associated with all three outcome variables. Wealthy women were 5.4 times more likely to make the recommended ANC visits by WHO, and 11.8 times more likely to deliver in health facilities compared to poor women. Variations were also found among employed women with regard to the use of the three outcome measures. Employed women were 75% more likely to make the recommended number of ANC visits and 38% more likely to attend PNC compared to unemployed women.

**Table 3:** Bivariate analyses of antenatal (ANC), delivery (FBD) and postnatal care (PNC) GDHS 2014.

Parameters	Antenatal	Delivery	Postnatal
	OR	OR	OR
**Individual level variables**			
**Socio-economic variables**			
*Education*			
No education	1	1	1
Primary education	1.05 (.149)	1.53(.120) ***	1.08 (.143)
Secondary/Higher education	1.76(.147) ***	1.29(.107) ***	1.12 (.119)
*Wealth status*			
Low income	1	1	1
Middle income	1.27 (.145)	2.17(.122) ***	.723(.126) ***
High income	5.47(.191) ***	11.8(.185) ***	.987 (.145)
*Employment status*			
Not working	1	1	1
Working	1.75(.127) ***	.908 (.112)	1.38(.099) ***
**Demographic and socio-cultural variables**			
*Age of respondents*	1.02(.001) **	.982(.001) ***	1.01 (.001)
*Ethnicity*			
Akans	1	1	1
Ga/Dangme	.475(.214) ***	1.08 (.210)	.864 (.229)
Ewes	.562(.199) ***	.765 (.191)	1.04 (.165)
Northern tribes	.733(.150) **	.579(.136) ***	1.97(.137) ***
Others	.485 (.397)	.299(.314) ***	1.11 (.305)
*Religious denomination*			
Christian	1	1	1
Muslim	1.32 (.162)	.821 (.150)	1.39(.139) **
Traditionalist	.608 (.263)	.222(.309) ***	.897 (.288)
No religion	.492(.219) ***	.384(.202) ***	.994 (.219)
*Marital Status*			
Never married	1	1	1
Married/Living together	1.73(.167) ***	.740 (.173)	1.15 (.143)
Divorced/Separated/Widowed	1.16 (.232)	.572 (.2310**	1.11 (.210)
*Residence*			
Urban	1	1	1
Rural	.423(.154) ***	.158(.149) ***	.857 (.179)
*Parity*			
Less than 3	1	1	1
3 to 5 births	.975 (.118)	.603(.085) ***	1.17 (.094)
6 or more births	.638(.154) ***	.424(.125) ***	.971 (.137)

*P<0.1

**p<0.05

***p<0.01

robust standard errors in brackets; OR = Odd Ratios

**Table 4:** Bivariate analyses of antenatal (ANC), delivery (FBD) and postnatal care (PNC) GDHS 2014.

Parameters	Antenatal	Delivery	Postnatal
	OR	OR	OR
**Access to healthcare**	* *		
*Respondent has NHIS*			
No	1	1	1
Yes	1.77(.117) ***	1.75(.095) ***	1.26(.097) **
*Distance to health facility (km)*			
Not a problem	1	1	1
A problem	.803 (.127)	.695(.087) ***	.814 (.143)
**Community level variables**			
*Residence*			
Urban	1	1	1
Rural	.991(.001) ***	.982(.002) ***	.998 (.002)
Years of education	1.21(.027) ***	1.44(.025) ***	.947(.029) *
Income score (Ghana cedis)	1.01(.001) ***	1.01(.001) ***	.998(.001) **

*P<0.1

**p<0.05

***p<0.01

robust standard errors in brackets; OR = Odd Ratios

In addition, ethnicity was associated with some of the outcome measures under consideration. Compared to the Akans and the Ga/Dangme, Ewes and Northern tribes were less likely to make the required number of ANC visits, but women from the Northern tribes were more likely to attend PNC compared to the Akans. Compared to Christians, Muslim women were 39% more likely to go for PNC. Likewise, Traditionalists were less likely to utilize FBD services compared to Christians. The analysis showed that majority (84.7%) of the women were married women or living together with a partner and they were more likely to attend ANC compared to divorced/separated (6.8%) or never-married women (8.5%). On the other hand, divorced/separated women were less likely to seek FBD compared with the never married women.

Rural dwellers were less likely to make the WHO recommended ANC visits and less likely to seek facility-based delivery compared to their counterparts in the urban centers. Parity was significantly associated with women’s use of ANC and facility delivery services. Women with four or more births were less likely to access health facilities for both ANC and delivery services compared to women with less than three births. Pregnant women with NHIS cards were more likely to access all three maternal health services compared to those without NHIS cards. Distance to health facilities was negatively associated with women’s use of ANC, FBD, and PNC services. Education was found to be a significant predictor of uptake of maternal health services at the community level. As indicated in Tables 3 and 4, communities in which women with higher average years of education resided compared with those with low average years of education, were significantly more likely to access ANC and deliver in health facilities.

### Multivariate analysis

Tables 5 and 6 summarize multivariate analyses; these examined the net effects of both individual and community-level factors on the three outcome variables. The multivariate results were largely consistent with some of the bivariate findings. Results indicated that women’s educational background was statistically associated with FBD (86%) and PNC (44%) services in Ghana after adjusting for other relevant variables. Also, the wealth status of households within which women resided, affected their decision to utilize maternal health services (see Tables 5 and 6). Ethnicity was statistically associated with women’s use of ANC, FBD, and PNC services. Regarding religion, Muslim women were more likely to make the recommended number of ANC (54%) visits compared to Christians, but Traditionist and women with no religious affiliation were less likely to use FBD and PNC services.

**Table 5:** Multivariate analyses of antenatal (ANC), delivery (FBD)and postnatal care (PNC) GDHS 2014.

Variables	Antenatal	Delivery	Postnatal
	OR	OR	OR
Individual level variables			
Socio-economic variables			
Education			
No education	1	1	1
Primary education	1.06 (.149)	1.23 (.124)	1.28 (.154)
Secondary/Higher education	1.26 (.165)	1.86 (.119) ***	1.44 (.141) ***
Wealth status			
Low income	1	1	1
Middle income	1.04 (.164)	1.30 (.136)	.788 (.133)
High income	3.04 (.278) ***	3.99 (.245) ***	1.13 (.189)
Employment status			
Not working	1	1	1
Working	1.71 (.143) ***	1.02 (.121)	1.33 (.104) ***
Demographic and socio-cultural variables			
Age of respondents	1.04 (.001) ***	1.02 (.001)	1.01 (.001)
Ethnicity			
Akans	1	1	1
Ga/Dangme	.394 (.234) ***	.984 (.199)	.856 (.229)
Ewes	.574 (.193) ***	.904 (.196)	1.09 (.167)
Northern tribes	1.17 (.186)	1.38 (.157) **	1.93 (.165) ***
Others	.511 (.403)	.471 (.328) **	1.18 (.319)
Religious denomination			
Christian	1	1	1
Muslim	1.54 (.187) **	1.06 (.170)	1.12 (.153)
Traditionalists	.781 (.256)	.363 (.325) ***	.826 (.291)
No religion	.653 (.212)	.554 (.210) ***	1.01 (.233)

*P<0.1

**p<0.05

***p<0.01

robust standard errors in brackets; OR = Odd Ratios

**Table 6:** Multivariate analyses of antenatal (ANC), delivery (FBD)and postnatal care (PNC) GDHS 2014.

Variables	Antenatal	Delivery	Postnatal
	OR	OR	OR
Individual level variables			
Socio-economic variables			
Marital Status			
Never married	1	1	1
Married/Living together	1.41 (.196)	.899 (.187)	.929 (.160)
Divorced/Separated/Widowed	1.02 (.253)	.758 (.250)	1.03 (.219)
Residence			
Urban	1	1	1
Rural	1.21 (.195)	.506 (.172) ***	.571 (.250) **
Parity			
Less than 3	1	1	1
3 to 5 births	.711 (.153) **	.672 (.111) ***	1.16 (.124)
6 or more births	.427 (.242) ***	.583 (.181) ***	.964 (.193)
**Community Level variables**			
Access to healthcare			
*Respondent has NHIS*			
No	1	1	1
Yes	1.68 (.124) ***	1.161 (.098) ***	1.21 (.101) **
*Distance to health facility*			
Not a problem	1	1	1
A problem	.973 (.125)	.842 (.091) *	.806 (.143)
*Socio-economic variables*			
Years of education	1.14 (.047) ***	1.21 (.043) ***	1.02 (.054)
Average Income score (Ghana cedi)	1.01 (.002)	.998 (.002)	.998 (.002) ***
**Variance component**	.684***	0.795	2.22
**Intra-Class Correlations**	17.20%	19.50%	40.30%

*P<0.1

**p<0.05

***p<0.01

robust standard errors in brackets; OR = Odd Ratios

Women from the Northern tribes were significantly more likely to seek FBD (38%) and PNC (93%) compared to Akan women. Consistent with the bivariate results, the multivariate findings showed that rural dwellers were less likely to use FBD (49%) and PNC (43%) compared to urban dwellers. Further, variables tapping access to health facility were statistically robust even after controlling for theoretically relevant variables in the multivariate analysis. Community-level results suggested that women in communities with higher education were significantly more likely to access ANC (14%) and FBD (21%). Also, women in wealthy communities were significantly less likely to access PNC.

## Discussion

Results presented in this study demonstrated that majority of women had four or more antenatal visits, and a large proportion of them delivered in health facilities. A significant proportion of women attended PNC visits within two months following delivery. This is consistent with previous studies that show that majority of pregnant women in Ghana seek ANC from health professionals, deliver in health facilities, and also seek PNC after delivery [4, 28]. Our finding of increased usage of FBD and PNC could be the result of various policies rolled out by the Ghana Health Services (GHS)/Ministry of Health (MOH) and its partners to ensure progress towards the attainment of the MDG goals 4 and 5. These policies included the Ghanaian Government policies of exempting pregnant women from delivery fees in all public and religious health institutions since September 2003, followed by implementation of free maternal health care policy in July 2008, as well as the free maternal health care policy under the NHIS. These progressive policies, introduced women in Ghana to a range of subsidized and comprehensive health care services, including ANC and FBD [3, 29,30] and they have significantly reduced MMR more than two-fold between 1990 and 2015 [2, 3]. Our findings are also in contrast to studies in other developing countries where uptake of ANC and FBDs have been reported to be very low compared to the use of maternal health services in Ghana. For instance, Yebyo et al. [24] indicated that 88% of expectant mothers in Ethiopia preferred home delivery to FBD with majority of them not making the required number of ANC visits recommended by the WHO. Our results also differ from a recent study in Bangladesh, which reported that about 95% of pregnant women, regardless of complications and potential danger signs, delivered at home with the assistance of TBAs [31].

Ethnicity was found to be significantly associated with some outcomes; for instance, women from the Northern tribes (38%) were 1.38 times more likely to seek FBD and 1.39 times more likely to seek PNC services, compared to the Akans (1.00). This finding is inconsistent with many studies on the uptake of maternal health services in the three Northern regions of Ghana [7, 18]. In the past, majority of women reported using the services of TBAs for child birth compared to the services of skilled birth attendants [7, 32]. In addition, the GHS/GSS [4], found that four in every five births in the Greater-Accra region (Southern Ghana) were delivered in a health facility compared to one in four births in the Northern region. Thus, the recent finding could be the continuous health education within smaller communities and the many CHPS compounds and zones in the Northern region, following the introduction of the free maternal health policy. The finding that women with higher parity are less likely to utilize ANC and FBD is consistent with the works of Anyait et al. [33] and Haque et al. [31] who have argued that women’s decision to utilize maternal health services is dependent on their previous experiences with labor. A possible explanation for this paradox is that women who have had four or more births may perceive themselves as experienced with the birthing process and thus, rule out the need for skilled attendants at birth. It is intriguing, however that women who were employed, were less likely to attend facility-based delivery. This counter-intuitive finding may be because we use an employment variable that is extremely limited. In their analysis of antenatal care in Ghana, Banchani & Tenkorang [34] used a detailed ‘employment’ variable to demonstrate that the majority of employed women in Ghana are in fact ‘self-employed’. They also noted that unlike women in ‘managerial/professional/clerical’ and ‘service ‘occupations, self-employed Ghanaian women tend to be less educated, leading to lesser use of ANC. Another possibility is that they are not able to take the time off to attend ANC since they are self-employed daily wage earners with limited income. Thus, the relationship between employment, education and maternal health outcomes for women in Ghana is complex and needs to be properly unpacked with qualitative studies. Interestingly, Kamal [35] found that employed and educated women were more likely to seek FBD and Caesarean section compared to unemployed and uneducated women in Bangladesh. Kamal further demonstrated that women from high socioeconomic status, patronize private health facilities for quality maternal health care services. Kamal’s finding is consistent with some studies in Ghana showing that women from higher socioeconomic status prefer the use of private health facilities during labor and PNC [32, 34]. In spite of the aforesaid explanations, the finding needs to be investigated further to better understand why employed women compared to the unemployed are less likely to access FBD services.

Our study confirms that rural dwellers are significantly less likely to use FBD services compared to urban dwellers. Besides issues of health infrastructure, women residing in rural areas lack financial resources to pay for transportation cost to go to a health facility beyond their catchment areas even when the cost of services are covered by NHIS. Other studies have also linked urban residence with higher quality of maternal health care services in Ghana compared to their rural counterparts due to the availability of modern health facilities [11,36, 37]. Access to NHIS remained a strong predictor of utilization of maternal health services even after controlling for socioeconomic and demographic factors. This is an indication that NHIS is helping bridge the inequality gaps in maternal health access.

It is well known that distance to the health facility is likely to affect the probability of pregnant women seeking care in most developing countries including Ghana [22, 24]. In Ghana, Gething and colleagues [19] stated unequivocally that greater the distance to a health facility, lower the chances of women using them in the rural areas, making it extremely hard for the majority of expectant mothers to seek health care. Their study showed that approximately a third of women in rural Ghana live more than four hours away from district health facilities and, therefore, are at higher risk of dying in the event of unexpected complications during labor or delivery. Other studies in Ghana and elsewhere have argued that distance to health facilities, lack of transportation, poor quality of care and the lack of information sometimes discourages poor women from seeking assistance from a skilled attendant during labor and delivery [24,38, 39].

A major objective of this study has been to examine if beyond individual-level predictors, there are some community-level factors that influence maternal health care access in Ghana. We found that women dwelling in communities with higher average years of education were more likely to utilize maternal health services. These observations are in consonance with past studies that highlight the positive impact of women’s education on maternal health services utilization [7, 10, 40]. This reiterates the debate on the significance of girl child education in developing nations in their quest to reduce maternal and infant mortality to an acceptable level. A higher level of education among women in a community changes the social dynamics and creates other social opportunities for women [41, 42]. Social norms may shift to favor better uptake of maternal health services, with a resulting improvement of pregnancy outcomes. Educated women also have the fiscal power and bodily autonomy to make decisions in times of emergency without waiting on their partners or in-laws which can avert the three delays, that result in maternal mortality and morbidity [43]. Moreover, continuous health promotion activities in the form of maternal education should be provided by health professionals periodically to inculcate maternal health care seeking behavior among women in such communities. Health professionals could strengthen this by partnering with local media houses (radio stations) to promote the importance of maternal health care services in various communities. For instance, health promotion in these communities during home visits could be undertaken to educate women and their partners on the importance of maternal health, the chances are that women would possibly utilize health facilities for its intended purposes.

Another important finding of this study was the negative association between wealth and the use of PNC services at the community level. Women in wealthier communities were significantly less likely to use PNC services. This finding differs from previous studies that indicated that wealthy women are more likely to seek immediate PNC due to the availability of funds to go to hospitals for deliveries and possibly PNC services. Although, women from wealthier households are more likely to benefit from media campaigns through radio and television on the importance of PNC compared to their counterparts from poorer households, the quality of care of post-natal services at the community level may deter wealthy women from seeking PNC services, provided by the state especially when there is lack of privacy and technical competency of the health personnel [44]; they can afford to use private facilities while the women from poorer communities rely on the state-provided PNC since they are either free or there is a modest fee. Other related studies have argued that the lack of awareness in the community about the significance of PNC services could make some mothers reluctant to patronize these services [45]. In the same vein, wealthy women in Ghana have the option to travel outside their communities to nearby urban centers to access quality maternal health services including PNC.

## Limitations of the study

Notwithstanding the findings of this study, its weaknesses must be considered. Data are cross-sectional, so we are unable to draw ‘causal’ connections between outcome and predictor variables. Our findings also do not apply to women who did not access ANC, FBD, and PNC while pregnant, so we do not have information on all women who conceived during the five-year period prior to the survey.

## Conclusions

Findings from this study revealed that women’s education and wealth status are highly associated with the use of FBD and PNC. NHIS was positively associated with maternal access at both the individual and community-levels with all the outcome measures. Geographical distance to health facilities was found to be negatively associated with maternal access. These findings have policy implications for health care delivery, especially in rural Ghana. We recommend that government of Ghana employs inter-sectoral collaborations to address the problem of long-distance to health facilities to ensure access to maternal health services to all women and improve access to female education.
